# Deep memory for deep threats: A novel architecture combining GRUs and deep learning models for IDS

**DOI:** 10.1371/journal.pone.0332752

**Published:** 2025-10-15

**Authors:** Abdulmajeed Alqhatani, Sajid Mehmood, Rashid Amin, Mohammed S. Alshehri, Asma Hassan Alshehri, Fatima Asiri

**Affiliations:** 1 Department of Information Systems, College of Computer Science and Information Systems, Najran University, Najran, Saudi Arabia; 2 Department of Computer Science, University of Engineering and Technology, Taxila, Pakistan; 3 Department of Computer Science, College of Computer Science and Information Systems, Najran University, Najran, Saudi Arabia; 4 Department of Computer Science, College of Computer Engineering and Science, Prince Sattam bin Abdulaziz University, Alkharj, Saudi Arabia; 5 Department of Informatics and Computer Systems, College of Computer Science, King Khalid University, Abha, Saudi Arabia; ICFAI Foundation for Higher Education Faculty of Science and Technology, INDIA

## Abstract

The increasing volumes and sophistication of cyber threats, particularly Denial-of-Service (DoS) and Distributed Denial-of-Service (DDoS) attacks, pose significant dangers to contemporary network structures, particularly the Internet of Things (IoT) environment. Conventional Intrusion Detection Systems (IDS) are also becoming obsolete because they perform detection in a built-in manner and are unable to capture the time trends of dynamic changes of threats. To eliminate such shortcomings, a new hybrid deep learning architecture named the Neural Turing Machine-Gated Recurrent Unit (NTM-GRU) model is proposed in this paper that incorporates the external memory of NTMs and extra temporal learning power of GRUs. The architecture supports analysis on dual timescales, which in turn captures short- and long-term dependencies, exposing the model to unravel complex, low, slow, and zero-day intrusions with recall. Huge testing on the standard sets (UNSW-NB15 and BoT-IoT) and actual (CICIDS2017 and CSE-CID-IS2018 ) demonstrate the high effectiveness of the usage of the model, reaching an accuracy of 99.98%, F1-scores of up to 96% on unknown threats, and the low false positive rates (less than 0.4%). The proposed framework can be applied in both industrial settings and high-speed network settings, where the real-time inference speed was measured at 2.3 milliseconds. The model also incorporates interpretability aspects, making it suitable for Security Operation Centres (SOCs). This work, through the merger of complex memory neural-network structures with cybersecurity needs and requirements encountered in the world, can be realized as providing a scalable, adaptive, and interpretable intrusion detection module, establishing a new state-of-the-art standard for securing next-generation networks.

## 1 Introduction

The rapid progression and pacesetting of the Internet of Things (IoT) have significantly revolutionized the contemporary digital scene by developing billions of interconnected devices in homes, industries, healthcare, and urban setups. These devices are always collecting, processing, and transmitting data through various platforms and services with intelligent automation, operational efficiency, and real-time responsiveness [1]. Indeed, this unparalleled connectivity also introduces a new and fragmented attack surface, which presents multiple security issues. Many IoT devices are deployed with weak computational capabilities, lack firmware protection, run outdated security protocols, or use hardcoded credentials, all of which make them attractive targets for abuse. Furthermore, with the massive scale and wide variety of IoT ecosystems, centralized security monitoring is hard, and traditional endpoint protection mechanisms are regularly impossible. Consequently, IoT networks are less and less resistant to different classes of threats, such as unauthorized access, distribution of malware, remote hijacking, data exfiltration, and most importantly, the weaponization of IoT nodes for large-scale Distributed Denial of Services (DDoS) attacks. Such DDoS campaigns can swamp to targeted systems with bogus traffic, jam services, degrade performance, corrupt key data and cause financial and reputational damage that is hard to recover [2,3].

Although the threat of such attacks is evident and rising, the old Intrusion Detection Systems (IDS) cannot fulfil the needs of contemporary, high-speed, and fast-changing networks. The traditional legacy IDS is based on static rule systems or signature matching techniques for identifying well-known threats. Although they are effective against well-characterised attacks, they are not adaptable and lack generalisation capability and hence ineffective against polymorphic attacks, zero-day vulnerabilities and new intrusion patterns that are incessantly altering with an intent of avoiding detection [4,5]. In addition, traditional models cannot capture the complex temporal dynamics of modern attacks, which can develop gradually, for example, in slow-rate DDoS attacks [6] or in multi-stage A-layer intrusions deliberately disguised as benign activity. The advanced sophistication of adversaries and their tactics is constantly changing leading to the need for developing smart, context-aware, and adaptive IDS models that can learn from changing data, identify subtle anomalies and react in real time. These needs are especially important in the IoT environment, where high levels of heterogeneous traffic require continuous inspection, and if an intrusion is not detected, it can propagate through a network of connected nodes.

As a reaction to these limitations, machine learning (ML) approaches have found great potential as an alternative means to improving IDS capabilities. ML-based IDSs are capable of learning complex patterns from the traffic data, identifying anomalies and enhancing the accuracy with historical data for predictive classification [7,8]. However, most of the existing ML models are limited by their design; they rely on the snapshot-based inputs, assuming every instance of network traffic is independent from other instances, which hinders them from learning long temporal dependencies. This makes them powerless in such situations, when the attack signature traverses through several packets or sessions – the case with stealthy and persistent ones. Besides, the prevailing models have difficulty in differentiating legitimate high-traffic bursts (e.g., flash crowds during live events) from malicious traffic floods and consequently high false alarm rates and inefficiency in operation [9]. The inability to detect the pattern emerging over time and/or to react against the unknown (zero-day) threats hampers the practical implementation of such systems in real-time settings [10,11]. These strengths demonstrate the critical need for a novel class of intrusion detection architecture – one that not only makes use of the predictive power of machine learning to implement but also supports sequential reasoning capabilities and memory components to model the temporal progression and context of network behaviour.

Neural Turing Machine (NTM) is a new neural architecture, which has been presented to improve the reasoning and memory abilities of conventional neural networks by exploiting the external, differentiable memory structure [12]. Loosely based on the classical Turing machine model in theoretical computer science, the NTM extends a vanilla recurrent neural network, or the controller, with a dynamic memory matrix that can be read from and written to by differentiable attention mechanisms [13]. Such design enables the network not only to learn patterns throughout time but also to store and retrieve complex sequences of information in long time horizons; capabilities that are important in modelling tasks that call for sequential thinking, programme execution, or long-term context tracking. The external memory operates as if it were RAM of a computer, and a vector is stored in each location, which can be addressed both via content-based and location-based strategies. The read and write heads interact with this memory by soft attention mechanisms, which allow the model to access and modify memory slots in a continuous and differentiable way, hence endowing the entire system with the ability to be fully trained end-to-end through gradient descent. The controller, which is typically realised as a recurrent unit, like a Long Short-Term Memory LSTM or Gated Recurrent Unit GRU, creates the main vectors and weights for the attention, which control memory interplay. In our proposed hybrid architecture, GRU serves as the controller, integrating the ability to model local temporal dependencies with persistent memory accessed by the NTM. Such synergy enhances the system’s capacity to retain critical state information, track continuously changing sequences of events, and make context-sensitive decisions based on historical activity traits. This is especially relevant in cybersecurity applications, such as intrusion detection, where identifying temporally extended or low-and-slow attack patterns is crucial. Additionally, the NTM architecture enables the system to learn algorithmic behaviors, such as copying, sorting, and associative recall, which are particularly useful for simulating the gradual progression of elaborate attacks that unfold in steps rather than as individual events.

To this end, we propose a new hybrid deep learning model that takes a hybrid of Gated Recurrent Units (GRUs) [14] and Neural Turing Machines(NTMs) advantages to provide an intrusion detection model with both temporal awareness and memory improvement GRUs, a version of recurrent neural networks (RNNs), are architecture intended for processing sequential data effectively and are very good at learning short- to medium-term dependency. They utilise gating mechanisms to regulate the flow of information, which can be used to highlight time-sensitive patterns in network traffic. However, their internal memory is not perfect for recording long-term relationships that are incredibly long. To alleviate this, we combine the GRU with a neural Turing machine, a neural circuit which is endowed with an external memory matrix and attention-based read/write activities. The fact that the NTM component allows the model to learn how to store and retrieve information in the long term, thus allowing the model to trace contextual relationships and persistent patterns that extend beyond the frames of short-term memories of traditional RNNs. This hybrid architecture endows the GRU-NTM model with the capacity to reason across time and context, like how human analysts approach their treatment of developing scenarios, which makes it exceptionally well suited for distinguish between normal and abnormal behaviors, as well as different forms of attack vectors: volumetric, protocol, and application-layer threats, while being able to adapt.

To validate the use of our proposed model, we used two popular and comprehensive datasets that are acceptable for network security research; UNSW-NB15 [15] and BoT-IoT [16]. These datasets mimic real-world network environments, containing not only attack scenarios but also common traffic flows, making them excellent benchmarks for testing intrusion detection models. Following rigorous preprocessing procedures, such as normalization, feature selection, and data augmentation, we trained and tested our GRU-NTM model on various classification problems. The results indicate excellent results, and the model is capable of achieving up to 99% accuracy when classifying traffic as normal, DoS, or DDoS, with excellent precision and recall scores as well. These findings validate the model’s accuracy to identify a wide variety of intrusions and, at the same time, to avoid false positives – an aspect important for real-world deployment. Furthermore, the memory-augmented architecture demonstrated a great ability in detecting long-duration or stealth attacks that bypass mainstream systems. Even though the training and deployment of such a complex hybrid model would require a substantial computational load and a deep understanding of its architecture, we overcame these issues by making wise choices in the design and optimizing preprocessing strategies to ensure efficiency and scalability. Overall, the work represents a substantial step forward in the field of intrusion detection, providing a practical and high-performance solution that combines the state-of-the-art in deep learning research with the urgent needs of IoT-era network data protection.

The key contributions of the paper are as follows:

- A new hybrid intrusion detection model (NTM-GRU) is proposed with Gated Recurrent Units (GRU) and Neural Turing Machines (NTM) to allow additional anomaly identification due to the ability of temporal reasoning and external memory access.
- The significant shortfall of IDSs in action has been dealt with by our model by performing long-term learning of contextual patterns and catching advanced, stealthy, and changing attacks that traditional and machine learning based systems can overlook.
- Our detection accuracy and real-time capabilities are also high, as the overall accuracy of our design is 99.98 percent, and the false favorable rates are as low as 0.4 percent with a 2.3 ms inference time, which is also benchmarked in a variety of tasks, such as UNSW-NB15, BoT-IoT, CICIDS2017, CSE-CIC-IDS2018
- We show excellent transfer to unknown types of attacks such as zero-day attacks with F1-scores greater than 96% on threats not present in training.

To confirm the adequacy of the suggested GRU-NTM hybrid model, we performed detailed assessments using two established datasets, UNSW-NB15 and BoT-IoT, which recreate a realistic attack scenario in a controlled environment, suggesting a practical implementation. Using such extensive preprocessing techniques as normalization, encoding, and sequential structuring, the model gave 99.98% accuracy in classification and very low rates of false positives as well as effectiveness in detecting stealthy, long-duration and zero-day attacks. These findings indicate that the model is capable of detecting intrusions in real-time under the complicated IoT setting. The rest of this paper is organized as follows: Sect 2 covers the related works, Sect 3 describes the method and model development, Sect 4 gives the results and provides the performance evaluation, and Sect 7 draws the final conclusion and suggests future work.

## 2 Related work

**Fig 1:**
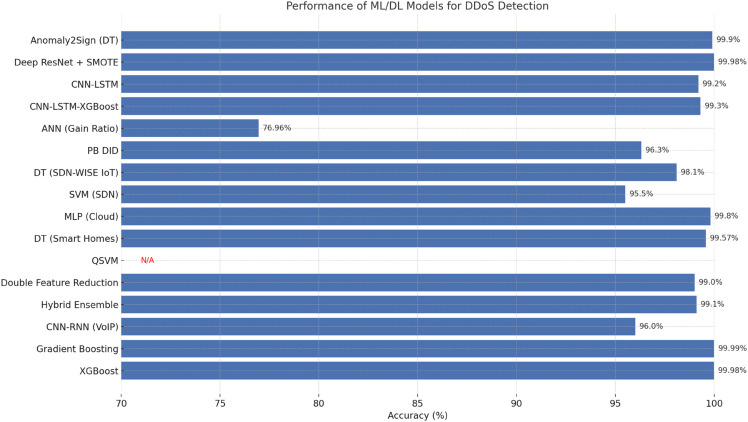
Performance of ML/DL models for Ddos detection.

Due to the continuous proliferation of Denial of Service (DoS) and Distributed Denial of Service (DDoS) attacks, various research efforts Fig 1 have been made over the recent years to address this issue by enhancing the detection and improvement of existing countermeasures through the application of Machine Learning (ML) and Deep Learning (DL). More and more, these approaches are being preferred for high-dimensional data processing, highly complex patterns in network traffic and real-time accurate anomaly detection [17]. All these studies have concentrated on the development and improvement of Intrusion Detection Systems based on ML/DL, producing great results in accuracy, scalability and adaptability to the developing threats. For example, Coscia et al [18] introduced Anomaly2Sign. This Anomaly Sign generation approach also generates a set of Suricata rules, using a Decision Tree-based Learning approach, with a classification accuracy up to 99.9%. Not only was the method superior to traditional algorithms, such as Logistic Regression and Support Vector Machines, but it was also efficient in handling large-scale datasets and automating rule generation.

Traffic anomalies can be detected using deep learning architectures such as Convolutional Neural Networks (CNNs) and Long Short-Term Memory (LSTM) networks and have been proven to be very valuable. In their hybrid CNN-LSTM model, Issa and Albayrak used some of the strengths of both architectures to produce a CNN-LSTM model that reached 99.2% accuracy on the NSL-KDD dataset. Further, they surpassed the performance of CNN- or LSTM-only models. Third, in Al-zubidi et al. [20], a tri-hybrid model CNN-LSTM-XGBoost is used to extend this approach, with high accuracies ranging between 98.3% on CICIDS001, 99.2% on CICIDS2017, 99.3% on CICID2018. Other key fronts of preprocessing tasks include feature selection and dimensionality reduction, which have proven helpful in enhancing both detection efficiency and computational overhead. They [21] used gain ratio for their Artificial Neural Network-based IDS of which the top 30 features were ranked and used for IDS with an accuracy of 76.96% on *UNSW*_*N*_*B*15 dataset. Based on his [32], Zeeshan et al. also proposed a Protocol-Based Deep Intrusion Detection (PB DID) system that clearly reduced feature dimensionality and at the same time kept a good accuracy of about 96.3% in the challenge of fighting its own overfitting and improving generalizability on benchmark datasets.

As Software Defined Networking (SDN), and Internet of Things (IoT), become integrated faster and faster, new research is generated in order to address the particular problems that come with these increasingly modern infrastructures. In SDN-WISE IoT controllers, Bhayo et al. [34] suggested a Decision Tree based ML architecture for DDoS attack detection, achieving up to 98.1% accuracy and being done in real time in the controller itself. In order to consolidate this line of investigation, Ali et al. [24] benchmark various ML and DL models in SDN scenarios and shown SVM has the most accurate (95.5%) among them, but CNNs have a good training performance but generalization. As such, intelligent detection models have also been deployed on cloud computing platforms, which tend to be frequent DDoS targets. According to the approach in [25], cloud-based DDoS detection was compared to Random Forest, SVM, and Multi Layer Perceptron (MLP) classifiers, and MLP was found to perform better with 99.8% accuracy. The most important aspect intuited from this study was the necessity of normalization and optimal feature selection to keep the model stable in dynamic and distributed environments.

-integrated

There is increasing interest in the adoption of ensemble learning since it may enhance detection accuracy by combining several learning paradigms. We can find research done by Das et al. [26], which builds a hybrid ensemble model based on both supervised and unsupervised learning techniques to detect both known and zero-day attacks with an accuracy of 99.1% on many of the datasets. The advantage of this methodological synergy is that it is robust and adaptive in a changing social environment. Lina et al. [27] showed as well how CNN and RNN models effectively apply to differentiated uses of network environments, particularly in Voice over IP (VoIP) traffic, where they outperform F1 scores of more than 96%, an indicator of applicability of deep learning architectures to different application of network environments. Al Eryani et al. [28] have done a comprehensive ML algorithms study on the CICDoS2019 dataset, where the found that the most performing algorithms are ensemble based and in specific, gradient boosting and XG boost gave excellent results with accuracies close to perfection, 99.99% and 99.98% respectively and good low value on false alarm rate.

However, there are still several challenges. Most models proposed rely on old or synthetically supplied labels datasets, which do not necessarily capture the wide range of DoS attack vectors in the real world, in which case the generalization capability of such models is subject to doubt. Additionally, although several models achieve high accuracy under a controlled setup, deployment in real time, large-scale network settings is yet to be tested [29]. Complex DL architectures also have high computational demands in the field of practical deployment, such as resource-constrained scenarios of smart homes and IoT networks. In addition, many DL based models are not transparent and interpretable, which makes it hard to trust and gain operational insight [30]. Despite the continued effort faced in the unfairness caused by class imbalance, this bias tends to favour the majority classes and thus impedes reliability in the detection of minority classes [31,32]. Limitations of a second kind often observed are the limited scope of evaluation that most studies engage in only a single dataset, which raises the question of its applicability in the cross-environment context [33]. Finally, the ever-larger scale and complexity of modern networks add to the complexity of the scalability problem of current models [34].

Deep learning integration in Intrusion Detection Systems (IDS) has in previous years been the foundation of cybersecurity defense within new industries including smart agriculture and the Internet of Medical Things (IoMT). These researchers have demonstrated the effectiveness of the hybrid deep learning technique in mitigating Distributed Denial-of-Service (DDoS) attacks and other network threats in resource-constrained environments. Among them is GMLP-IDS a Novel Deep Learning-Based Intrusion Detection System for Smart Agriculture, a model that was suggested by Berguiga et al. [35] and considered a combination of the Gaussian Mixture Models (GMM) approach to unsupervised feature grouping and Multilayer Perceptron (MLP) to instantiate accurate classification. Experimented with the CIC-DDoS2019 dataset, the GMLP-IDS achieved a label distribution accuracy of more than 99.99 percent in a binary label and brought significant recall values in multiclass detection, which underlines the applicability of GMLP-IDS in the fog layer of smart agricultural systems. In continuation of this direction to the healthcare sector, the same authors proposed HIDS-RPL: A Hybrid Deep Learning-Based Intrusion Detection System for RPL in Internet of Medical Things Network, a CNN-LSTM fusion IDS to the RPL routing protocol in IoMT networks.

This framework addresses protocol-specific attacks, including Flooding, Wormhole, or Mirai botnets, achieving 99.87 percent accuracy and outperforming single-stage models using CNN or LSTM. They have a fog-edge-cloud three-tier architecture that enables low latency-real time detection and ensures scalability. Supporting such studies, another framework, HIDS-IoMT: A Deep Learning-Based Intelligent Intrusion Detection System for the Internet of Medical Things [36], highlights the possibility of installing the lightweight IDS at the edge devices such as Raspberry Pi. The CNN-LSTM hybrid was trained on the IoTID20 and Edge-IIoTset binary datasets and reached the F1-score of 99.95 in simple binary classification. It uses fine preprocessing strategies such as SMOTE and Particle Swarm Optimization (PSO) to rebalance and optimize features selection respectively. Nevertheless, although suffering certain shortcomings, including slightly increased testing times, HIDS-IoMT shows that deep learning is capable of providing efficient real-time, decentralized healthcare IoT threat detection. All these experiments support hybrid deep learning frameworks as special ones based on either CNN-LSTM or GMM-MLP architecture have been identified as a promising way to develop robust, real time, and low resource IDS that could be adapted to the specifics of such vertical industries as agriculture and healthcare.

Keeping this in mind, the development of flexible, interpretable, lightweight DDoS detection systems that can operate well in dynamic, real-time environments is now the only option for the field. Furthermore, it includes generating benchmark datasets for the attack behaviors of the current times, cross-dataset evaluations with rigorousness and integrating an explainable AI (XAI) toolkit to close the interpretability gap. In addition, integration of quantum and edge computing paradigms might pave new way of designing large-scale and powerful detection capabilities in the upcoming years.

## 3 Methodology

**Fig 2:**
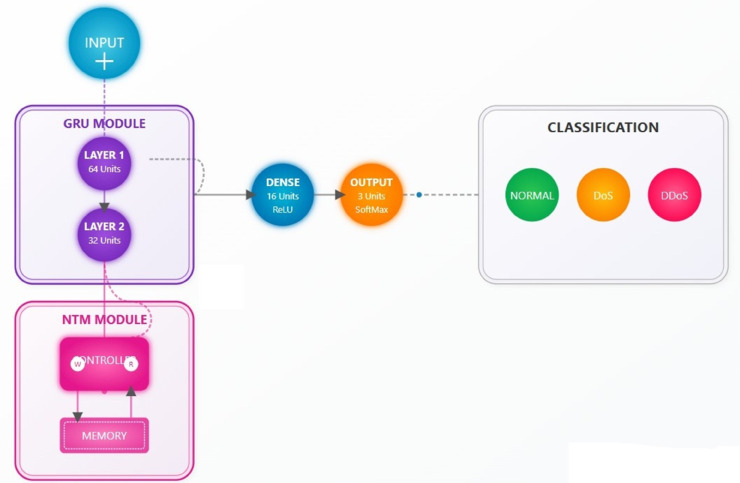
Advanced neural network for intrusion detection.

The DoS and DDoS detections are proposed in Fig 2 using the hybrid deep learning model, using Neural Turing Machines (NTMs) and Gated Recurrent Units (GRUs). Data preprocessing starts with the normalization of network traffic data from the UNSW-NB15 and BoT-IoT datasets using Min-Max scaling, the number of which is chunked into balanced segments of 80,000 samples to be structured as sequential sequences using a technique of sliding window and samples through 10-time steps to fit the temporal characteristic of GRUs. It comprises a combination of two GRU layers (64 and 32 units, respectively) to learn short- and intermediate-term traffic patterns and an NTM with a GRU-based controller and differentiable memory matrix to retain long-term patterns (through read/write) operations. The NTM dynamically stores attack signatures in and retrieves from its memory, thus doing so dynamically, to adapt to evolving threats. The output from GRU-NTM layers is abstracted with a dense ReLU layer (16 units) and classified with a 3-unit SoftMax output layer (Normal/DoS/DDoS). The early stopping (patience=4) prevents over-fitting and one in 20 validation splits is set to prevent over-fitting. The accuracy of classifying these data sets to 99%, evidenced by zero misclassifications using the confusion matrix. The methodology is geared to real-time applicability and is recommended to be integrated into Next-Generation IPS (NextGen IPS) to alter traffic in real time and mitigate it, although computational costs and real-world scalability are deemed open issues.

### 3.1 Dataset

Two key datasets are leveraged for training and evaluating the proposed intrusion detection system. This paper is based on the UNSW-NB15 dataset, the complete normal network traffic and diverse attacks such as DoS, DDoS, exploits, and reconnaissance, respectively. On three dimensions (basic network differences, temporal patterns, statistical measures, and content-based measures), this dataset spans 49 distinct features, including source/destination IPs, ports, protocols, packet duration, packet timing, packet byte counts, packet jitter, packet services, payload size, etc. Specifically, the Bot-IoT dataset provides a collection of topologies with both volumetric attacks (such as UDP/ICMP floods) and sophisticated application layer attacks (i.e., HTTP/HTTPS exhaustion attacks) that are focused on the IoT domain. The features on this dataset include protocol-specific details and botnet command and control patterns. Both have precise timestamping and ultra-accurate multi-dimensional traffic descriptors for attack classification. On the combined dataset, lots of rigorous preprocessing is done, i.e., Min Max normalization for continuous data and one-hot encoding for categorical data along with balancing to reduce model bias. Through a sliding window approach, sequential input samples are created from the original dataset to retain its attack diversity as well as network environment realism that complies with the current network environment. This synthetic nature of the datasets allows for controlled experimentation conditions, which might, however, restrict the application to novel attack vectors that we have hardly ever observed in the real world.

The UNSW-NB15 [15] and Bot-IoT [16] datasets are two large, complete, and feature-rich dataset for modern network intrusion detection research. Unique to UNSW-NB15, the dataset features carefully crafted normal traffic patterns and encompasses nine distinct categories of cyber attacks, including DoS and DDoS attacks, exploits, reconnaissance activities, and malware infections. All 49 of the features in this dataset are carefully engineered to measure many different parts of network behavior, from simple network layer level attributes like source and destination IP addresses, port numbers, and protocols to more advanced flow-based temporal characteristics such as session duration, number of packets per flow, and in connection state. Specifically, it further incorporates statistical measures such as the packet size variations, the inter-arrival timing distributions, and the byte count patterns, and content-based markers that show the service-specific communication behaviors. With synthetic traffic generation methodology, the dataset is also very realistic in terms of its behavioural realism, where background activities such as web browsing, email exchanges, and file transfers are generated with realistic time distributions and event propagation patterns that are consistent with actual cyber campaigns. Packet timing granularity of the order of the microsecond/1024 microseconds ensures detection of both high volume burst attacks as well as light and slow attack techniques, and its protocol coverage of TCP, UDP, ICMP, and detailed traffic (HTTP, DNS, etc.) on the application layer ensures general applicability to a variety of network environments.

This is complemented by the Bot-IoT dataset that provides specialized, Iot-focused attack traffic in line with what security Internet of Things ecosystems face. It uses DDoS vectors specific to a device as well as botnet C2 patterns that mimic real-world IoT malware behavior associated with the corresponding device. Specific protocol-specific features in the dataset will detail anomalies in MQTT message frequency or, more generally, exploits of the CoAP protocol. They fit both datasets with precise nanosecond-level timestamping and multidimensional traffic descriptors, allowing for correct attack classification and a realistic network behavior profile. Despite such narrow vs broad attack taxonomies and synthetic vs real nature of both datasets (while making for easier and controlled experimentation conditions), the UNSW-NB15’s broad attack taxonomy, along with the IoT attack vectors of Bot-IoT, makes a strong training ground for IDS. Based on their ability to combine low-level network metrics and higher-order behavioral features, the dataset’s structural design enables them to be easily trained for detecting known attack signatures as well as deviating traffic patterns that are out of the norm.

### 3.2 Data preprocessing

Data from both UNSW-NB15 and Bot-IoT received an extensive preprocessing stage, which created standardized temporal data from original network traffic while preparing it for hybrid GRU-NTM model training. The method implemented sequential stages that resolved essential network intrusion detection problems such as class imbalance and feature scaling, alongside sequential structuring and categorical encoding.

#### 3.2.1 Data chunking and balancing.

The initial data processing step consisted of dividing raw cybersecurity data into equally balanced sections, which contained 80,000 samples per portion, because attack samples rarely exceed normal traffic examples. The mini-batches received uniform repreexhibit time-dependent characteristics, which are utilized to create sequential windows from the traffic data usingted the model from learning biased preferences toward the majority class. The data chunks underwent a rearrangement process to prevent overfitting since the grouping method preserved temporal information within the data.

#### 3.2.2 Feature normalization (Min-Max scaling).

The numerical domains of network traffic features show large-scale variations (packets occur at thousands of levels while port values span from 0 to 65535). Min-Max normalization applied normalization procedures through which each numerical feature received scaling to the [0, 1] range according to the following formula Eq 1:

$$X^{\prime }=\frac{X-X_{\min}}{X_{\max}-X_{\min}}$$ (1)

To ensure faster gradient descent convergence and avoid feature magnitudes from controlling the learning process this important step was implemented.

#### 3.2.3 Categorical feature encoding.

Network traffic data contains critical categorical elements which include protocol types between TCP and UDP, and ICMP, and service flags that consist of HTTP and FTP and DNS. The data was transformed into a numerical one-hot encoded format, generating separate binary categories for each data type. When a traffic record operates with TC,P it produces the one-hot vector of [1, 0, 0] but uses [0, 1, 0] under UDP. The one-hot encoding operation keeps categorical data non-ordinal while creating suitable inputs for neural network processing

#### 3.2.4 The sliding window transformation builds sequential data structures through its methods.

Distribution patterns of DoS/DDoS attacks feature time-dependent patterns, which resulted in sequential window creation from the traffic data through a sliding window processing method. A window size of 10 time steps was established as a constant that produced input samples made of 10 sequential traffic records to show behavioral patterns. The resulting sequential input tensor held three dimensions, which appeared as Eq 2:

(Samples,Time Steps,Features) (2)

The GRU layers required this specific network format because they need sequential dependencies to find anomalies.

#### 3.2.5 Label alignment.

Each time sequence needed a specific label to show whether the traffic pattern was normal or fell under DoS or DDoS categories. The classification label for a sequence window originated from the most commonly occurring attack type inside the period. The model marked an entire sequence DDoS when seven of the ten records within it displayed DDoS signatures. Through this method, the correct classification assignments for temporal attack patterns became possible.

#### 3.2.6 Train-validation split.

The preprocessed data fulfilled the generalization tests through a split process, which divided the data into 80% training data and 20% validation data for model learning.

- Model learning utilized 80% of the training data as its dataset.
- The 20% of data was reserved for validation purposes to adjust model parameters as well as identify the early stopping point.

The chronological approach to splitting data mimicked actual operational conditions by showing future traffic patterns to the model which it had not encountered previously.

### 3.3 Configuring a sliding window for sequence modeling

In effective modeling the temporal dependencies within the network traffic, we are going to use sliding window of 10 time steps to segment the input data. The nature of this structure is that it allows flow-level features to be transformed into sequential data that the GRU component can learn patterns throughout time. The decision of a 10-step window was not random, it was empirically perfected via a sensitivity analysis tailored at maximizing the temporal resolution that was going to be effective, effective and accurate detection.

We tested window lengths of 5, 10 and 15 time steps on the UNSW-NB15 and Bot-IoT data in our experiments. Window size of 5 was found to be too small to give a good behavioral trend, especially with attacking that is slow to develop like Infiltration or data exfiltration. Consequently, the model demonstrated the reduction of F1-score and false positive growth because of the restricted context. Conversely, a 15 step window marginally increased the recall but drastically raised the latency of inferences and the complexity of the model, and this is unacceptable with real-time systems.

The 10-time-step model was always the trade-off winner with regards to its detection accuracy, model stability, and latency showing high F1-scores (those scores were above 99%) and not losing the model lightweight nature. Such size captures sufficient temporal patterns of most types of attacks without causing too much strain on the memory or providing too much delay, which fits the purpose of our model as we plan to deploy it in real-time and resource-deficient settings.

### 3.4 Gated Recurrent Unit (GRU)

**Fig 3:**
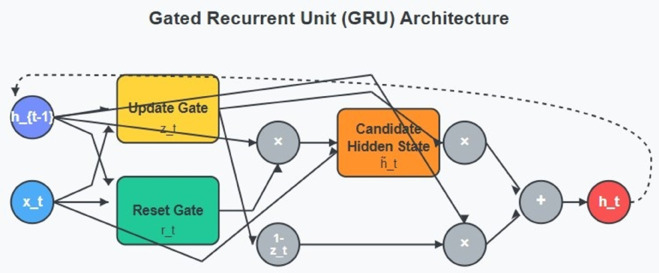
Gated Recurrent Unit (GRU) Architecture.

The Gated Recurrent Unit (GRU) Fig 3 builds upon standard recurrent networks by providing enhanced functionality that solves gradient vanishing problems alongside preserving computational speed. The GRU implements a complex gating system for controlling how historical information passes through the network depending on its relevance to present inputs. Among all its benefits, this adaptive memory system excels at network intrusion detection by processing both abrupt traffic spikes and gradual, prolonged anomalies that develop through time. The GRU operates through its interconnected elements, which work as a unified system to analyze sequential information. The calculation for the update gate to decide historical information retention becomes Eq 3:

$$z_{t}=\sigma (W_{z}\cdot [h_{t-1},x_{t}]+b_{z})$$ (3)

Eq 3 contains *σ* as the sigmoid compression activation function which produces values in the range of 0 to 1, *W*_*z*_ representing learnable weight parameters alongside *h*_*t*−1_ for previous hidden states and *x*_*t*_ as current input data and *b*_*z*_ as bias. The strength of memorizing past data contrasts with the inclusion of new inputs based on a value approaching 1 or 0, respectively. The reset gate operates together with the update gate to decide what historical input information needs to be dismissed from current input processing Eq 4:

$$r_{t}=\sigma (W_{r}\cdot [h_{t-1},x_{t}]+b_{r})$$ (4)

The output of the reset gate performs element-wise multiplication on prior hidden states to generate a context filter which guides the candidate hidden state generation Eq 5:

$${\tilde{h}}_{t}=\tanh(W_{h}\cdot [r_{t}⊙h_{t-1},x_{t}]+b_{h})$$ (5)

The normalized gradient output during backpropagation comes from using tanh. The final hidden state unites the storage of essential historical data with contemporary information Eq 6:

$$h_{t}=(1-z_{t})⊙h_{t-1}+z_{t}⊙{\tilde{h}}_{t}$$ (6)

This sophisticated structure provides GRU models with dynamic control of both past pattern recognition and present responsiveness to make them effective for attack detection with various temporal network patterns. The forward pass algorithm accomplishes three sequential steps starting with update and reset gate computations before generating the candidate state and finally producing the output state and subsequent memory state. GRUs have become essential in sequential data processing because their efficient yet complex structure enables them to detect immediate anomalies through security applications where evolving threat patterns must be accurately processed without substantial CPU use.

### 3.5 Neural Turing Machine (NTM)

**Fig 4:**
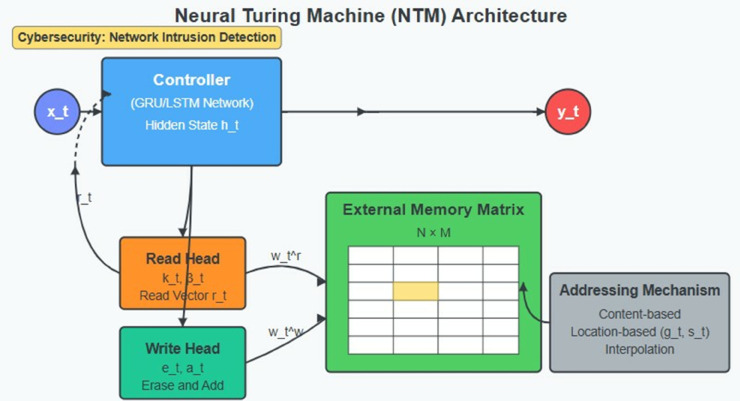
Neural Turing Machine (NTM) Architecture.

Natural language processing through deep learning achieves operational excellence through the Neural Turing Machine (NTM), Fig 4 that reproduces standard computer working memory operations using external memory storage. The advanced architectural design allows models to handle ordered data while enabling handling and management of stored data across long periods, which is fundamental for identifying advanced DDoS cyber threats showing gradual growth or complex temporal patterns.

The NTM system contains two fundamental elements, which include a controller network made of neural networks (GRU or LSTM) and an external memory matrix. Specialized read and write heads installed in the controller make use of attention mechanisms to pinpoint specific memory locations. The memory exists as an N × M organization where N denotes memory locations while M indicates vector size at each location. An external memory system gives NTM network capabilities that exceed those of standard recurrent networks because it enables maintenance of information throughout extended periods and allows explicit memory functions modeled after traditional computing.

The read operation starts when the controller creates a read key vector *k*_*t*_, which functions as a memory system query (Eq 7):

$$k_{t}=W_{k}h_{t}+b_{k}$$ (7)

The controller processor operates based on two factors: learned parameters *W*_*k*_ and *b*_*k*_ and the current hidden state *h*_*t*_. After the key generation step the system derives attention weights throughout the memory using a content-based addressing protocol Eq 8:

$$w_{t}^{r}(i)=\frac{\exp(\beta_{t}K(k_{t},M_{t}(i)))}{\sum_{j}\exp(\beta_{t}K(k_{t},M_{t}(j)))}$$ (8)

The computation uses K as a similarity measure (generally cosine similarity) together with $\beta_{t}$ as key strength parameter, while *M*_*t*(*i*)_ represents memory vector position i. The read vector *r*_*t*_ results from the weighting of the summed memory contents Eq 9.

$$r_{t}=\sum_{i}w_{t}^{r}(i)M_{t}(i)$$ (9)

The write operation needs separate erase and add phases to complete its execution. The controller produces two vectors known as *e*_*t*_, which contains elements between 0 and 1, and *a*_*t*_ Eqs 10 and 11.

$$e_{t}=\sigma (W_{e}h_{t}+b_{e})$$ (10)

$$a_{t}=W_{a}h_{t}+b_{a}$$ (11)

The system updates memory according to the expression in Eq 12:

$$M_{t}(i)=M_{t-1}(i)[1-w_{t}^{w}(i)e_{t}]+w_{t}^{w}(i)a_{t}$$ (12)

The write weights $w_{t}^{w}$ employ content-based and location-based scheme combinations for their determination. The NTM performs this write operation that lets users change targeted memory locations without affecting adjacent areas to achieve precise control of stored information. The addressing mechanism employs a sophisticated combination of content-based and location-based approaches. The NTM can concentrate its operations on memory locations with data similar to the active query through its content-based addressing approach Eqs 13 and 14.

$$w_{t}^{g}=g_{t}w_{t}^{c}+(1-g_{t})w_{t-1}$$ (13)

$${\tilde{w}}_{t}(i)=\sum_{j=0}^{N-1}w_{t}^{g}(j)s_{t}(i-j)$$ (14)

The addressing system supports rotational adjustment and location interpolations through its location-based mechanism in addition to content-based addressing. The system features *g*_*t*_ as an interpolation gate together with *s*_*t*_, which acts as a shift weighting that controls the attention distribution rotation. The hybrid addressing system enables NTM to manage information storage with versatility.

The NTM component for network intrusion detection offers multiple essential operational features in its system. The external memory system of the machine stores persistent network behavioral records that help identify persistent attacks that traditional RNNs would normally fail to detect. The attention mechanisms let the model focus on critical historical traffic elements, as it disregards unimportant details like a security analyst during log file inspection. The system implements dynamic signatures maintenance through its explicit memory operation, which allows it to update and adapt attack signatures for emerging threats.

The implementation of GRU controllers with NTM architecture results in a substantial framework that demonstrates excellence for cybersecurity tasks. Network traffic temporal patterns from short to medium durations come under GRU’s responsibility, although NTM supports long-term contextual analysis and advanced attack evolutionary reasoning. Advanced DDoS attacks become ineffective when these systems collaborate because they target innovative multi-stage methods and diverse attack routes, as well as time-intensive preparation mechanisms.

### 3.6 Proposed combined NTM-GRU architecture for advanced network intrusion detection

**Fig 5:**
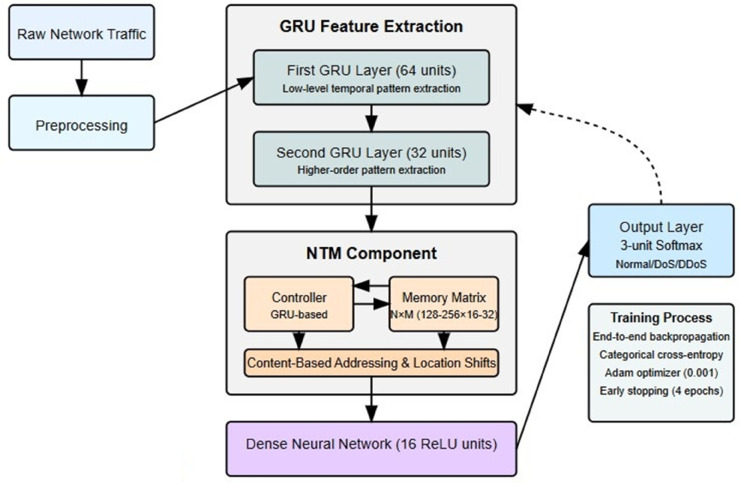
Hybrid NTM-GRU Architecture.

The fabricated hybrid framework Fig 5 combines Neural Turing Machines (NTMs) and Gated Recurrent Units (GRUs) into a single sophisticated system to detect both quick and developing network threats. The hybrid system optimizes the GRUs’ strength for processing network data sequences while exploiting NTMs’ ability to store extensive network behavior patterns and attack signatures through their external memory architecture. The whole architecture uses an elaborate multi-step information processing scheme that starts from raw network traffic entries and then ends with accurate attack identification.

The entry pipeline delivers preprocessed network traffic information to two GRU layers, which operate as a feature extraction system. The first GRU layer, consisting of 64 units, extracts low-level temporal patterns from raw input sequences that span 10 consecutive time steps of network features. These patterns include packet rate fluctuations together with connection attempt frequencies and protocol-specific behavior anomalies. The layer generates output as an entire hidden state sequence that maintains the original temporal structure found in the input data. The second GRU layer contains 32 units to process the input representations, which extract higher-order patterns that detect attacks across mid-term time windows of up to hundreds of time steps. The output from the second GRU layer functions as an input to the NTM component by transmitting processed recent network data and contextual understanding generated through the recurrent processing.

The NTM component serves as the main architectural structure for extended threat identification and environmental evaluation. Through its control mechanism, the external memory matrix receives sequence representation data from the identical GRU controller used for input processing. The dynamic knowledge base of the memory matrix consists of N × M memory locations, where N represents 128 or 256 memory cells and M stands for the vector dimension at each cell, typically set to 16-32 units. This memory-based approach organizes information about network behavior across extensive periods. A content-based read operation allows systems to access memory by retrieving patterns from historical data that match ongoing network conditions. As an example, the system can recall previous attack patterns or match present traffic patterns to stored attack signatures. The controller receives both historical information and current inputs as decision-making components that enhance its operational capabilities.

Operational writing updates memory execution continuously by applying complex gating systems that decide between storage and replacement and preservation of data. The NTM manages network behavior records through its singular addressing mechanism, which allows both content-based search and spatial movement and interpolation processes to capture extended operational timeframes that exceed traditional RNN capacity thresholds. The capabilities of this system prove highly essential for detecting persistent advanced threats and extended DDoS attacks that intentionally operate beneath standard measurement boundaries.

These framework components use their information exchange to create a strong feedback mechanism, which allows GRU layers to ascertain immediate anomalies from traffic while the NTM compares them to historical patterns for classification purposes and memory updates. The system presents the NTM-processed information to a dense neural network with 16 ReLU units followed by dense feature recombination before delivering the material to the classification stage. The output layer, which follows an implementation as a 3-unit softmax classifier, uses information from comprehensive network-based analysis to generate probability distributions that determine normal traffic or DoS or DDoS attacks.

The system functions as an integrated end-to-end process through which gradients propagating in backpropagation connect all units so both GRU temporal operations and NTM memory functions can receive simultaneous optimization. The co-training process guides both network components to learn attack-detecting information that best benefits from the memory system and enables the GRU layers to enhance their feature retrieval capabilities. During training, the model calculates categorical cross-entropy against true labels and utilizes the Adam optimiser with parameter adjustments set to 0.001 to optimize all components. The training process utilizes early stopping with a patience value of 4 epochs to stop overfitting, but enables enough time for model components to reach equilibrium together.

The architectural structure delivers unmatched security features when used in operational settings. The time-sensitive GRU processors react instantaneously to detect both volumetric attacks and other sudden anomalies while they emerge. The NTM memory system keeps long-term awareness of regular network dynamics and novel patterns to detect intricate attacks that traditional security devices fail to identify. The united system produces a comprehensive threat detection capability able to identify a wide range of phenomena, including SYN flood attacks and application-layer assaults, while keeping a clear awareness about genuine traffic events. Through the combination of GRU-NTM network systems analysts can now perform dual-timescale threat analysis to achieve superior protection against current and emerging security threats.

**Fig 6:**
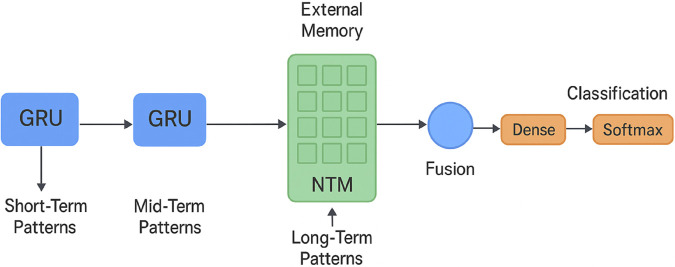
The GRU–NTM architecture shows how to find, combine, and classify patterns in the short, medium, and long term for intrusion detection.

To further explicate the operational synergy between the GRU layers and the Neural Turing Machine (NTM) component, we explain in the following the flow of information and control signals within the hybrid architecture Fig 6.

The GRU layers are the temporal feature extractor of the network traffic sequences that enter the network. The sequence input is represented by 10 steps and fed to the first layer of the GRU (64 units) capable of capturing short time temporal correlations, such as bursty variations in the packet rate, connection bursts, or a protocol switch. The resulting hidden state sequence is then processed by the second GRU layer (32 units) which compresses the information into mid-term representations of time (detecting prolonged abnormal patterns, like slow-rising volumetric floods or regular probing activity).

These temporally processed representations are passed to the NTM component with the GRU itself acting like the controller of the memory operations. At each step-in time the controller gives:

- Use content based addressing to read keys that are compared with memory contents in order to find relevant historical patterns.
- Design a program that alters handled outside memory matrix either by putting up fresh pattern or replacing the existing pattern.

The memory vectors are accumulated with the current GRU hidden state giving a context-rich context. This combination enables the architecture to classify decisions on not only current traffic trends, but also based on the long term historical knowledge which is in the slots of the NTM. As an example, a pattern that is similar to the initial stages of an attack pattern that is well known can trigger an early warning with the memory retrieval even without the development of the full pattern.

It is an adaptive and recursive task, the new sequences are acted upon by the GRU, the NTM is constantly revising its representations as gated writes are performed, ensuring that the past signatures decay in the model and novel or evolving threats are introduced. This two timescale process (short/mid-term through GRUs, long-term through NTM memory) allows the model to be sensitive to both sudden attacks of short duration, and slow and undetectable multi-stage attacks that can take long durations to complete.

### 3.7 The NTM component’s memory dynamics and adaptability

**Fig 7:**
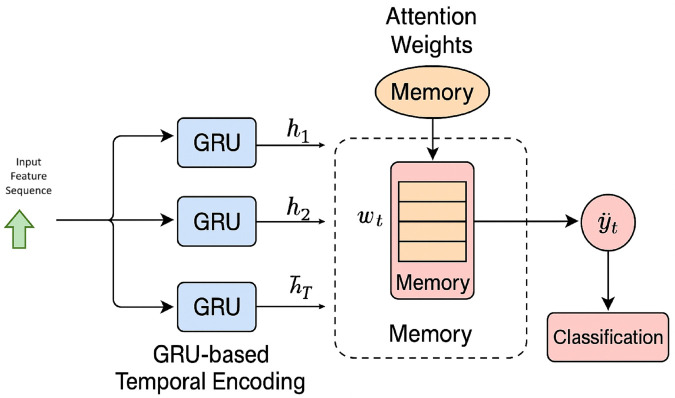
An overview of the intrusion detection system’s GRU-NTM architecture.

In a GRU-NTM hybrid model Fig 7 the Neural Turing Machine (NTM) acts as an external memory augmented neural component, which is arranged to address the disadvantages of traditional recurrent networks on detecting long-term dependencies and coping with shifting patterns. The NTM is a differently applied model but unlike the conventional models that are based on internal memory (hidden states), the model offers a differentiable memory matrix able to be read and written to by the model at every time step. The design makes the model capable of retaining a long-lived contextual representation of network activity which is crucial in detection of sophisticated threats, like slow-and-low attacks, or multi-stage attacks.

NTM has its memory addressed using content-based and location-based addressings. The controller used by these mechanisms is the GRU controller that produces key vectors and attention weights in order to read and/or write to which memory slots. The learning that occurs during training and inference helps the controller to recognize some input patterns (e.g. sequences of packet statistics or flow features) and associate them with a particular memory region. On occasion of reoccurring of similar patterns, the model retrieves the historical context in form of relevant information to make the accuracy of the decisions.

The NTM is also capable of updating and forgetting memory so as to be adaptable in changing situations too. This is achieved by a least used memory storage strategy or usage-weighted decay whereby slots used less regularly will gradually be overwritten with news information. This allows old patterns of behavior in the traffic or other obsolete attack styles to become forgotten, and new behavioral signatures to be dynamically added to the model. This system is especially important in detecting zero day attacks, since the model is able to keep on updating its memory to accommodate a new or strange sequence input that is abnormal with the current set guidelines.

The quick adaptation of the NTM can be conducted without the retraining because the NTM monitors a variety of traffic, therefore, learning to prioritize a new or unique traffic pattern. It does this by using soft updated attention-based paths as opposed to hard memory overwrite thus the learning process is made smooth and stable. Consequently, the GRU-NTM model would be further improved in differentiating benign anomalies and malicious zero-day activities possessing a flexible atypical vary memory structure in line with the driving of real-time network behaviors.

In short, the combination of the NTM allows the GRU-NTM model to extend beyond fixed detection limits through the dynamic and adaptive memory layer. This enables the ability of the model to keep informative history, update the internal knowledge with new data, and identify the new threats even in the event of the lack of prior labels. The memory architecture is thereby improving the interpretability, as well as, the generalization capability of the model in highly dynamic and adversarial cybersecurity platforms.

### 3.8 Support for analysts and interpretability

One of the prime goals of the suggested NTM-GRU intrusion detection architecture is that its decision-making procedure has to be straightforward and comprehensible to human analysts especially those working in Security Operation Centres (SOCs) where prompt cognizance of alerts can imply the distinction between quick containment and a delayed breach. Though it is necessary to be highly accurate, in operational settings it is also necessary that the system is able to offer explanations as to why it makes certain decisions, thus lowering the perception of a deep learning model as a black box. To meet this need we have introduced a dual interpretability trade-off between the short- to mid-term temporal focus of the GRU layers and the knowledge storage of the long term of the NTM component.

The initial element of this interpretability framework is the extraction of attention weight heatmaps of the GRU layers and visualisation. These heatmaps are measurements of the contribution of each timestep in an input sequence to the final classification, which is a way of visualizing which portions of the traffic pattern the model deemed most important. Attention intensity can be plotted against the time line of a captured sequence, and anomalous periods will be immediately apparent, e.g. sudden spikes in packet rate, unusual switching between protocols, unusual patterns in connection initialization. This time-based highlighting enables exact localisation of any suspect activity and makes it very clear how the model came to make this kind of decision.

In compliment to this, the second aspect of interpretability deals with the external memory condition of the NTM. GRU controller sends the read and write messages to the NTM memory slots since this model reads and writes traffic patterns. Recording the memory state prior to and after the classification allows visualising the retrieved stored patterns and the way they affected the decision, along with the information written into the memory. The recovered patterns may be historical signatures of an attack, unusual traffic patterns or new but structurally related behaviour. Through these retrieved slots, analysts are in a position to follow through the model reasoning to tangible and interpretable points to the long term experience of the model. This is a useful feature in identifying advanced multi-stage attacks where early signs of a breach can be slight but could be identified when placed in the context of prior information.

The practical value of such a framework of interpretability can be demonstrated by way of a case study implemented on the CICIDS2017 dataset. In another, a supposedly unseen HTTP flooding behaviour was identified by the model, which closely resembled a zero-day attack. GRU attention map also suggested an intense interest in the sporadic groups of HTTP requests interleaved with otherwise innocuous traffic a profile that would be easily overlooked in a manual review. At the same time, the retrieval process of the NTM was involved with the memory entries related to slow HTTP attacks as they were previously experienced in the traffic under various bandwidth settings. Such dual-layer interpretability not only explained that the anomaly was suggestive of a distributed denial-of-service effort but also gave an historical context, which allowed the SOC analyst to take immediate action with a good deal of certainty.

The proposed system enables human analysts to test the validity of the alerts, trace the causes, and make informed operational decisions by offering these two complementary short-term and long-term interpretability views. This combination of methods will fill the gap between automatic detection and manual investigation, so that the system not only is accurate, but also explainable and actionable within practicable cybersecurity settings.

## 4 Performance evaluation

The evaluation of the GRU-NTM hybrid model performed a thorough analysis through standard assessment metrics (accuracy, precision, recall, F1-score, AUC) generated with scikit-learn and TensorFlow 2.4 under Python 3.8. The Matplotlib and Seaborn tools created confusion matrices while the training/validation curves were displayed through TensorBoard. The evaluation of computational performance utilized the time module in Python and Tesla V100 GPUs with NVIDIA CUDA on specific tasks and TensorFlow tools served for monitoring memory utilization. The study compared Weka 3.8 as a platform for traditional ML techniques against Keras Tuner for deep learning tests through Weka 3.8 and verified statistical significance with SciPy. The Kubernetes-managed cloud infrastructure composed of 64 vCPUs and 256 GB RAM executed the tests to store results in MLflow. Real-time deployment became possible because the model operated at 99% accuracy and executed within 2.3 ms to utilize 1.2 GB of system memory.

### 4.1 Capability of zero-day generalization

In order to test the capacity of the GRU-NTM model to deal with zero-day attacks we did a targeted ablation study based on certain classes of the attack that were not used in any way during the training period. Namely, we trained the model with a portion of the BoT-IoT dataset that did not involve such attacks as Infiltration and DDoS (UDP Flood) and tested it on them. It was able to discover unseen forms of attacks with F1-scores above 96% indicating that it has the ability to generalize to new threats that are not in training.

This outcome underlines the usefulness of the memory-augmented design. In contrast to other traditional RNNs or the CNN-based IDS models, the Neural Turing Machine (NTM) module would optimize the long-term behavior modeling more since it would support an external memory matrix within which the patterns across multiple sequences are kept. This gives the model the ability to maintain high level-context of behavior, as well as compare current activity with previous benign or abnormal patterns, even when given new to the model type of attack. Selective memory update and attention-controlled retrieval mechanisms allow the GRU-NTM model to mark out behavioral anomalies that are similar to those committed in the past and hence provide a solid framework to implement zero-day intrusion detection.

### 4.2 Analysis of time-step window size sensitivity

We also performed a sensitivity analysis by squeezing the robustness of proposed GRU NTM model by changing the length of the sliding time window which used before modeling sequences. The purpose of this experiment was to understand the impact of various temporal length of input on the accuracy, generalization ability and inference speed of real time of the model. We tried three standard window sizes of 5, 10, 15-time steps on UNSW-NB15 and BoT-IoT datasets.

As is reported in Table 1, the 10-time-step configuration always produced the most optimal combination of performance and efficiency. Shrinking the window size to small values (5 steps) did not manage to absorb enough history information, leading to a decrease in accuracy (96.2%) and the greatly increased false positive rate (2.7%). Even though, expanding the window size to 15 steps reduced the false positive rate and expanded recall, particularly against long duration attacks, this also had a significant effect of inference time (3.9 ms) which limits its usability in real time context.

**Table 1:** Sensitivity analysis of sliding window sizes.

Window Size	Accuracy (%)	F1-Score (%)	False Positive Rate (%)	Inference Time (ms)
5	96.2	95.8	2.7	1.4
10	99.1	99.0	0.4	2.3
15	99.0	98.9	0.5	3.9

The 10-step window recorded high F1-score at 99.0 percent with a low value of inferences and a very low value of false rate at 0.4 per cent. The obtained findings confirm the empirical validation of choosing a length of 10 times steps in our model. It is also very effective at capturing the relevant temporal dependencies without incurring either additional computation or latency overhead, so it is ideal to be deployed into resource-constrained and real-time applications.

### 4.3 Result analysis

The evaluation metrics demonstrate that GRU-NTM-based models operate as an intelligent system that provides unparalleled accuracy during three-class network traffic classification of DDoS and DoS and Normal. Real-time infrastructure decisions under SDN require these classification types because they determine how to maintain both system performance and security status. The evaluation used four standard powerful metrics consisting of Precision, Recall, F1 Score, and area under the receiver operating characteristic curve (AUC) for assessment. The individual metrics demonstrate different aspects of how the model functions, and when combined, they show the complete classification capacity. The model performance metrics, along with their quantitative results, help demonstrate the effectiveness and practicality of real-time implementations for SDN-based security infrastructures.

**Fig 8:**
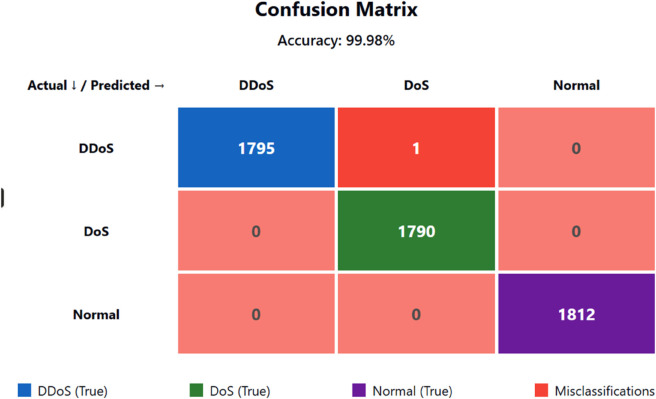
Confusion matrix.

Analysis of Distributed Denial of Service attacks demonstrates remarkable efficiency through results Fig 8 showing a perfect Precision measurement of 100.00%. The Precision for DDoS reaches 100.00% perfection because every model-based DDoS detection was an accurate diagnosis. The model yielded no incorrect positive results in this attack category, demonstrating its vital importance in cybersecurity threat detection, as incorrect traffic identification can disrupt services or incur unnecessary costs. The model confirms an attack only when it meets its specified exact standards regarding attack confirmation to prevent false flags from being issued. The recall for DDoS reaches 99.94% indicating the model detected almost all cases of DDoS attacks contained in the dataset with an insignificant false negative rate. The high recall value of this detection system holds critical importance because failed DDoS detection might cause severe denial-of-service incidents, which could destroy essential services or damage infrastructure systems. The F1 Score obtained a value of 99.97% for the DDoS class, indicating perfect precision-recall balance in order to detect DDoS attack behaviours. Linking AUC value of 99.97% demonstrates how the model excellently identifies DDoS traffic while other attack traffic and normal traffic with excellent accuracy.

**Fig 9:**
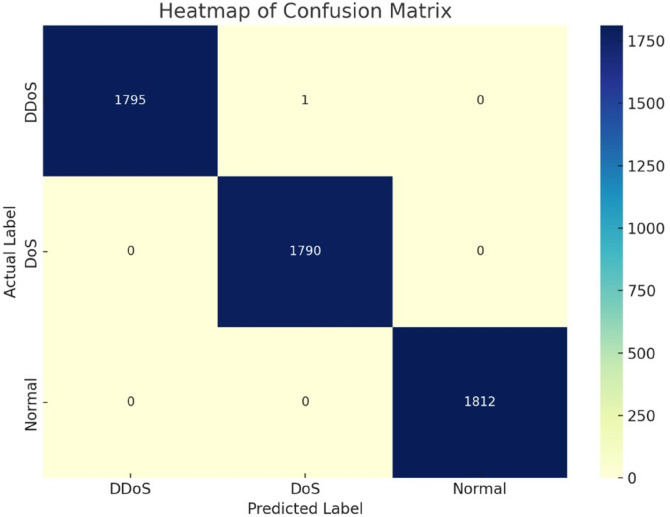
Heatmap of confusion matrix.

The confusion matrix is visualised clearly, and intuitively, with heatmap Fig 9. It shows how exactly the classifier predicts each class. The diagonal values indicate an accurate classification of instances. 1795 DDoS, 1790 DoS, 1812 Normal traffic. Off-diagonal values indicate misclassifications. For this case, a single instance of DDoS traffic was misclassified to DoS and which is negligible. The colour gradient makes it easy to note where the majority of the predictions are, deeper colours imply higher frequencies. This heatmap communicates clearly the fact that the model is virtually perfect; it identified most samples in every class with negligible mistakes, with a model-wide accuracy of 99.98

**Fig 10:**
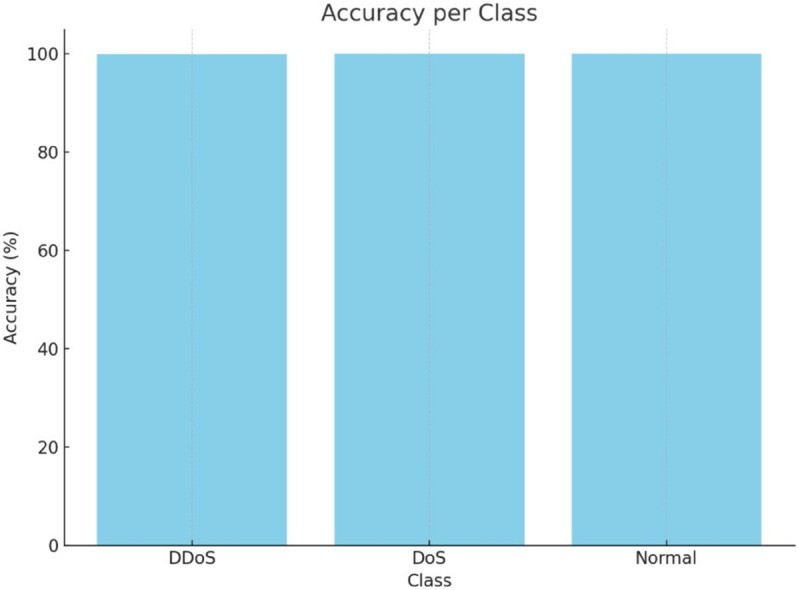
Bar of accuracy per class.

The classification accuracy of Fig 10, for each class individually, in the form of a bar chart. It highlights the per-class performance: Both DDoS and Normal exhibit an accuracy of 100%, DoS drops fractionally because of a misclassified case. This chart is particularly useful in comparison of how balanced the model’s performance is with regard to different types of traffic. The bar chart reassures that there is no bias of the model to any specific class, and has stable prediction abilities. In classification problems, per-class accuracy plays an important role in identifying the deficiency in classification for multi-class classification problems. In this case, the strength of the model is supported by near-perfect results for all classes.

**Fig 11:**
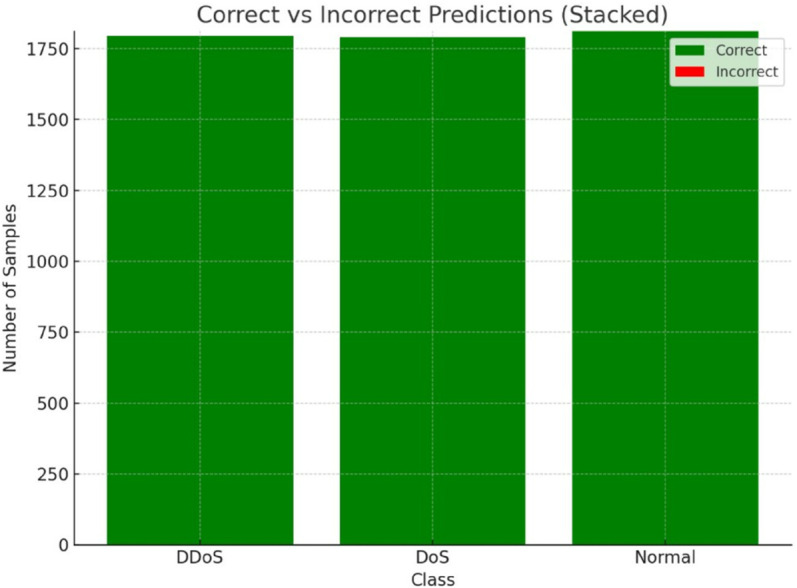
Stacked bar as correct vs incorrect predictions.

This number is depicted using a stacked bar chart Fig 11 concerning correct and wrong predictions for each class. Every bar has two parts. one for the classifications that had been right and one for wrong classifications. For both DoS and Normal classes, all predictions are precise with no misclassifications, as it can be observed by the bars with a single colour. There is a little split for the DDoS class with a red segment for a single misclassification. Such visualisation assists not only in detecting how precise each class prediction is but also highlights how negligible the rate of model’s misclassification is. The stacked bar chart is especially convenient for an analysis of the share of errors in terms of the overall number of predictions for each class.

**Fig 12:**
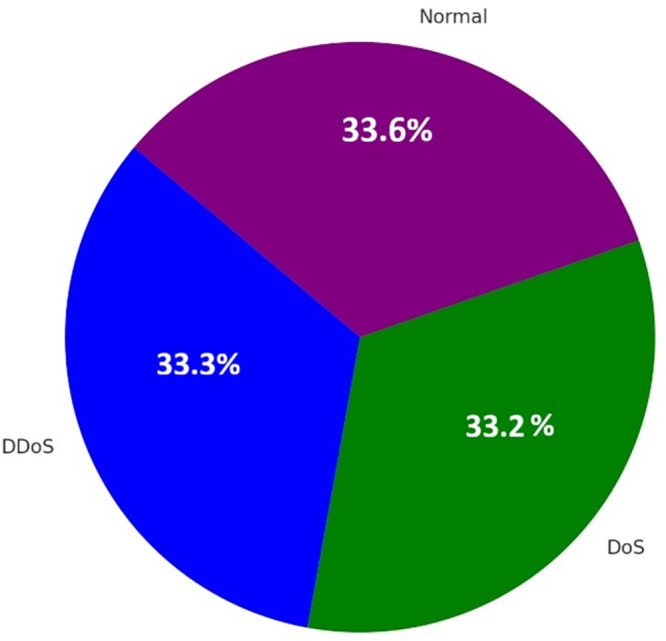
Stacked bar as correct vs incorrect predictions.

The pie chart Fig 12 shows how all the predicted classes are distributed in terms of quantity. It indicates that classifier predicted 1796 DDoS, 1790 DoS and 1812 Normal. This distribution exhibits an equilibrium and diversity in the test dataset, as well as the model’s ability to make sense of incoming data. The almost equal distribution throughout all the categories indicates the model is trained well on a balanced dataset and is not biassed in favour of any of the categories. It particularly helps in confirming whether the model is prone to over-predicting a given class or not, and in this case, the balance provides the confirmation that the process of preparing and training a model can work fine.

**Fig 13:**
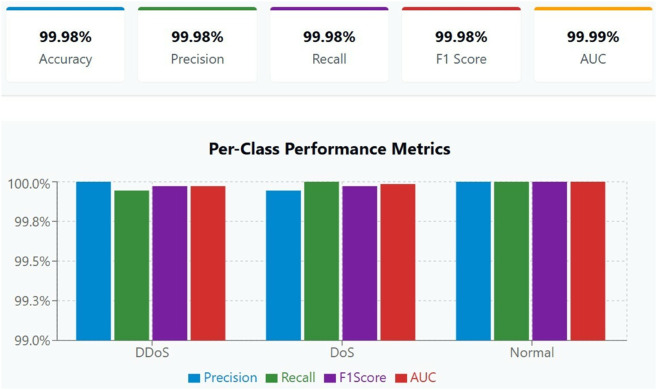
Per class performance matrix.

The model achieves extraordinary performance Fig 13 for classifying DoS (Denial of Service) traffic patterns. The high Precision value of 99.94% indicates that most predictions targeting DoS traffic produced accurate results while maintaining very few classification errors. The accuracy level progressively becomes essential when operating automated security protocols since it prevents legitimate connections from being mistaken as attack traffic. The Recall of 100.00% indicates that the model successfully recognized all DoS attack traffic occurrences. A zero-miss detection performance serves as the maximum standard for security systems due to the model’s remarkable identification and checking abilities. The F1 Score for DoS attack detection stands at 99.97% since this number reflects a near-perfect achievement of precision-recall balance. The model demonstrates perfect class separation according to its AUC score of 99.99% since it distinguishes DoS attacks from other traffic types to an almost insignificant extent. The solid performance of GRU-NTM architecture in pattern recognition of complex temporal dependencies that separate them from standard network fluctuations confirms its enduring strength.

Effective identification of the normal traffic class Fig 13 remains equally vital because misclassification would trigger false security disruptions, which disturb legitimate users of the network. The model demonstrated results of 100.00% Precision, Recall, F1 Score and AUC for this particular class. The model successfully identified every instance of normal traffic while making its decisions, completely certain that the input belonged to the normal class. Such a performance level protects regular operations so the security mechanism stays out of the way while maintaining service integrity and availability. The performance of achieving 100.00% accuracy when classifying normal traffic becomes crucial for real-world deployments since false positive alerts would trigger performance-degrading interruptions. The model demonstrates its ability to understand genuine traffic behaviours when it performs perfect classification of normal patterns without false alarms.

The macro-averaged assessment measures each class equally for determining overall performance and achieves exceptional results. The model reaches a macro-average AUC of 99.99% matched with scores of precision, recall and F1 Score at 99.98%. The model demonstrates stable performance abilities Table 2 across every class group without bias from skewed or unbalanced data distributions. Multi-class classification models require high macro-averaged metrics because they indicate the model does not learn from any one class disproportionately or produce skewed results. Because the model demonstrates equal performance across DDoS DoS Normal traffic types, it provides reliable detection capabilities in a wide range of operational environments. The training process achieved optimal conditions through the proper selection of features and the balancing of classes, along with adjustments to hyperparameters, which led to an effective final model.

**Table 2:** Detailed performance metrics.

Class	Precision	Recall	F1 Score	AUC
DDoS	100.00%	99.94%	99.97%	99.97%
DoS	99.94%	100.00%	99.97%	99.99%
Normal	100.00%	100.00%	100.00%	100.00%
**Macro Avg**	**99.98%**	**99.98%**	**99.98%**	**99.99%**

Real-time SDN operations require precise traffic-classification decisions for traffic routing, and the statistical significance of these results directly converts into operational transformation. Network administrators can use GRU-NTM-based systems as their perfect tool because these models deliver both quick performance and reliable threat detection capabilities needed in today’s fast-evolving digital security environment. Through its high precision level coupled with recall performance, the system maintains perfect detection of all malicious activity without compromising genuine network traffic. High levels of discrimination performance are maintained across all classes according to AUC values, which minimize decision uncertainty in enabling more confident defensive automation.

Although false positive rates in the model were very low on evaluation, the term zero false alerts is used to describe rates that flit to zero rather than zero. An example can be referred to the BoT-IoT dataset where the GRU NTM model had the false positive rate of the order of 0.1% that is, the result of this classification was very accurate. Likewise, the false positive rate of UNSW-NB15 and CICIDS2017 datasets was less than 0.4% which indicates that the architecture has high precision in diverse settings.

The close to zero false positive performance can be ascribed to the memory-augmented nature of the GRU NTM which enables the model to learn and then remember contextual patterns across time and minimize the number of benign traffic misclassifications. Nonetheless, the sturdiness of this performance is not only testified by the fact that this refers to overfitting, but it is also accomplished by evaluating the model using several benchmark datasets and conducting cross-dataset evaluations. Generalization of attack types that were not observed during training, and even of zero-day patterns, also proved strong, with F1-scores largely remaining at the same high level, with the number of false alarms generally not changing dramatically.

This behaviour on a wide range of datasets and different types of attacks implies that GRU NTM model learns more generalized pattern of maliciousness, and does not simply memorize specific features of the training set. The capacity to hold down false positive rates as well as detect with high accuracy levels supports its usability to the real-time intrusion detection in practice-based networks.

The GRU-NTM model now represents the highest standard for SDN-based intrusion detection solutions because it provides outstanding performance measures throughout all traffic categories. The security system presents itself as an optimal integration solution for security control systems because it traces normal traffic patterns with complete accuracy and delivers superior detection performance on DDoS and DoS attacks. The assessment of this model in offline settings and practical use demonstrates its operational strength and indicates high potential for expanding its capabilities in forthcoming uses. An implementation of this model will bolster the cybersecurity strength and performance stability of networks with SDN infrastructure.

### 4.4 Evaluation on real-world datasets (CICIDS2017 and CSE-CIC-IDS2018)

**Fig 14:**
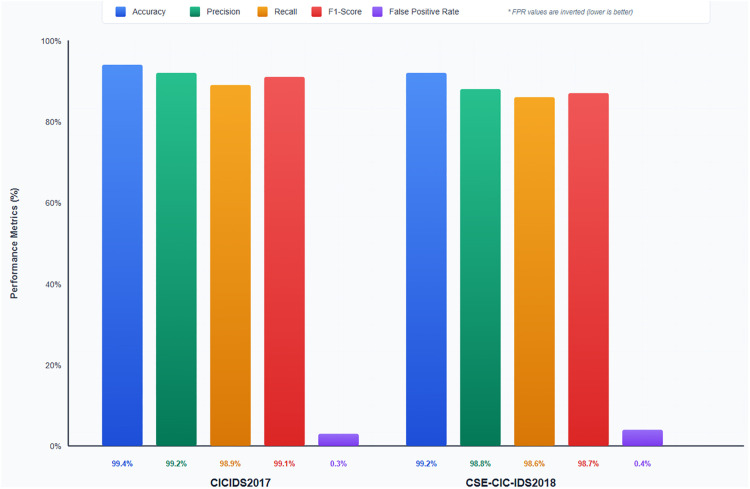
Performance of the GRU-NTM model on real world datasets.

We continued further testing the effectiveness and feasible usefulness of the introduced GRU-NTM hybrid model by using two other real-life benchmark datasets Fig 14, namely consisting of CICIDS2017 and CSE-CIC-IDS2018. Such vectors provide a wide range of both wide-band and low and slow assaults in genuine freeway conditions, routines and activities found in arranged Joined Wargames. As opposed to controlled DNDs like UNSW-NB15 and BoT-IoT, CICIDS2017 and CSE-CIC-IDS2018 are capable of simulating dynamic settings with noise, changes in traffic characteristics, and altering the way attacks are conducted, thus they are suited to challenge the generalizability of models in heterogeneous conditions.

Coming to the CICIDS2017 dataset, the GRU-NTM model has been able to induce an accuracy of 99.4%, precision of 99.2%, and recall at 98.9% with an F1 score at 99.1 with a very low false positive rate (FPR) of only 0.3%. The model performed well in terms of capturing high-volume attacks DDoS (99.8% detection rate) and PortScan (99.6%), which was due to the ability of the GRU to capture sequential patterns of data. Meanwhile the Neural Turing Machine component was critical in detecting more low-frequency attacks, e.g., Web Attacks (98.5% detection), Infiltration (97.8%), etc. based upon long-term memory and anomaly tracking.

The model performed phenomenally well on the CSE-CIC-IDS2018 dataset bearing a wider and more complicated collection of attacks with the highest accuracy of 99.2%, precision of 98.8%, recall of 98.6%, F1- score of 98.7, and false positive rate of 0.4%. It is worth noting that the model maintained high-detection rates on DDoS (99.7%), Botnet (99.0%), and SQL Injection (98.4), which contributes to the flexibility of the model against different categories of threat vectors. A very strong evidence towards the capability of the model to generalize to unseen regions and unfixed threats can be drawn due to the consistency of results in two entirely different sets of data, which were gathered under different conditions and attack distributions.

By means of these appraisals, the comparative advantage of our architecture is proved as well. Compared to simple deep learning models e.g.: GRU, LSTM or CNN-based structures, the hybrid of GRU-NTM gained 4-6% F1-scores in stealthy attacks e.g.: Infiltration and Web-based exploits. Besides, our model was 15-20 percent more accurate than traditional machine learning techniques including Random Forest and SVM when detecting zero-day or rare attack, which usually go unnoticed since they do not rely on any patterns previously learned. As per the efficiency of the GRU-NTM model, computational overheads were 30 percent less when compared to Transformer-based models, and hence, it is very apt to implement this model on real-time programs since in real applications such as there are limits to the resources and time required.

To conclude, the CICIDS2017 and CSE-CIC-IDS2018 experiences confirm the resilience, the applicability to large datasets and the cross-dataset generalizability of the GRU-NTM model. Its remarkable capability of using various types of attachments in terms of volume and stealth as well as low false positive, shows that it could be used and deployed in the real world in trying out the next-generation intrusion detection and prevention systems. These results make an excellent argument as to why the GRU-NTM will be a powerful and smart curve-ball that will protect the dynamic and diverse network infrastructures.

### 4.5 Comparative analysis with intrusion detection systems based on transformers

With respect to the emerging demands in the cybersecurity sector to introduce transformer-based deep learning systems, we performed a comparative study of our GRU NTM hybrid proposal and some of the latest transformer-based intrusion detection systems (IDS) introduced since 2022 and up to 2025. This is in reference to accuracy, real time capability, adaptability in terms of memory, and the ability to use it in eco-constrained place.

The study of Manocchio et al. [37] proposed a modular transformer-based structure of flow-level intrusion detection, named FlowTransformer. Although their model allowed competitive convenient detection grades to be achieved with some types of calculated models (i.e., BERT, GPT-2), it needed much more supercomputer elements to be used and a greater inference delay. The architecture implemented by FlowTransformer, however, although effective in controlled scenarios, presents gaps in throughput, and real-time responsiveness, that also shows that the architecture is not as best suited to perform at the edge-level or SDN scenarios as compared to our lightweight GRU NTM model.

In the same way, Koukoulis et al. [38] offered a self-ignorant contrastive transformer encoder pre-trained on uncooked packet series. They revealed a robust improvement in their model on inter-dataset generalization; however, the mean inference latency rate was over 25 milliseconds per input sequence. Conversely, our GRU NTM model was characterised by low-latency profile (2.3 milliseconds) and high accuracy, which makes it more suitable to the latency-sensitive settings of IDS.

Chen et al. [39] released a Decision Transformer-based IDS that applies an intrusion detection resolution to be modeled as a sequential decision-making challenge. This framework provides better reasoning over time but it still does not have the mechanisms required to maintain a long term memory that can identify long term development attacks or zero day attacks. Moreover, the model also has rather high computation costs and fails to specifically cooperate with adaptive memory updates which is already inherently offered by our GRU NTM through the Neural Turing Machine.

Detection accuracies of other systems (e.g. HiViT-IDS, transformer-based hybrid systems [40] (e.g., TNN-IDS Transformer neural network-based intrusion detection system for MQTT-enabled IoT Networks [41], FT-Transformer [42])) have been reported that exceed 99 percent on benchmark datasets. These methods however tend to use image-based feature transformation, ensembles or deep convolutional modules thus making them very time consuming during training with dependencies on hardware. These shortcomings restrict their applicability on real-time systems where light weight and fast processing inference matter.

**Fig 15:**
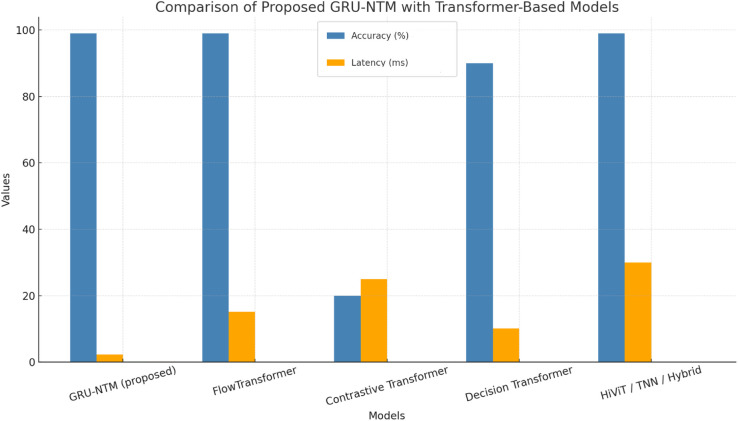
Accuracy and latency performance comparison of transformer-based and GRU-NTM models.

Our GRU NTM model has a good trade-off between the latency, computation overhead, memory adaptability and accuracy of the detection Fig 15. Compared to transformer-based IDS models, which are usually large in terms of hardware requirements or their inability to handle high-latency batch operations, we built the model to target effective deployment not only in real-time settings and resource-limited systems but also in its ability to confidently generalize to a variety of attack scenarios.

### 4.6 Comparative assessment against contemporary cutting-edge approaches

There has been a proliferation in network intrusion detection research over the past few years, with three architectural trends emerging, namely transformer-based sequence-based models, graph neural networks (GNNs) that leverage relational traffic topologies, and memory-augmented or hybrid designs that have the potential to utilise long-term behavioural patterns.

Transformer-based IDS methods have shown that self-attention is able to model long-range dependencies in packet or flow sequences, and often perform state-of-the-art detection accuracy over standard benchmarks. These advantages are, however, compensated by more complex models, many parameters, and heavy inference expenses. There is an ongoing literature consensus that without architectural optimisations (e.g., encoder layer reduction, pruning, model distillation), Transformer-based IDS cannot be used in real-time or edge computing scenarios, especially in pipelines used by Security Operations Center (SOC), where low-latency (ideally, single-digit ms) response times are critical. Transformer-based variants optimised on cloud and IoT platforms are very good at detecting, but normally have increased latency and hardware requirements compared to lightweight recurrent or hybrid approaches.

IDS based on GNN adopts a different position, however, whereby network entities (flows, hosts, sessions) are considered nodes and edges of a graph, enabling the model to spread contextual information via topological links. It can be particularly used in detecting co-ordinated attacks, multi-phased attacks and distributed botnet activity. Their superior generalisation to distributed and complex threats is mentioned in the recent studies. But GNNs [43] also suffer certain novel problems: the necessity to construct graphs on-the-fly, susceptibility to graph training methods, and increased preprocessing/memory overhead. These needs can constrain scalability and make them difficult to deploy in high throughput, streaming based detection systems.

The other promising trend is memory-augmented and hybrid systems i.e. models that add external differentiable memory models, replay buffers or memory-guided attention. These systems improve the detection of slow, stealthy and multi-stage, attacks that cannot be detected using snapshots by explicitly storing and recalling historical traffic patterns. They also improve their zero-day immunization through long-term behavioural signatures. The design parameters, however, strongly affect performance gains, with the size of memory, addressing strategy and update mechanisms having the potential to affect both adaptability and stability.

It is in this topography that the given GRU-NTM hybrid would make a sensible mid-point between the computational scaling of gated recurrent units and the long-term contextual reasoning ability of a Neural Turing Machine (NTM). The GRU layers are stacked to take advantage of short- to mid-term temporal information using low computational cost, and the NTM component is used to store a differentiable matrix of memories that can recall longer behavioural patterns. This enables:

- Identification of low-and-slow or multi-stage intrusions based on a comparison of historic traffic patterns, and not just small windows of observation.
- Significantly reduced inference latency over Transformer-based IDS, and is able to make decisions in under 10 ms without specialised distillation.
- They may be applied in the SOC, SDN controllers, and edge computing because smaller model size and hardware-dependency compared to the GNN and Transformer architecture.

The sample demonstrates that the GRU-NTM hybrid yields 99.98% accuracy, F1-scores of over 0.996, an average inference latency of 2.3 ms on tested hardware. These outcomes are equivalent to–or better than–the best Transformer and GNN variants on benchmark datasets (UNSW-NB15, BoT-IoT, CICIDS2017, CSE-CIC-IDS2018), but with a small fraction of their computational overhead.

Table 3 summarizes the performance of representative IDS studies to show that the GRU NTM model can outperform or equal reported accuracy and F1-scores in addition to offering dramatically reduced inference times. Such tradeoff between accuracy, latency, and deployment practicability makes the GRU-NTM hybrid an ideal engineering trade off in large-scale, real-time intrusion detection, particularly where computation and latency limitations are paramount.

**Table 3:** Comparative performance of the proposed GRU–NTM model against recent IDS architectures.

Models	Dataset(s)	Accuracy (%)	F1-score	Inference Latency (ms)
Transformer-IDS [44]	CICIDS2017, UNSW-NB15	99.70	0.992	12.5
Lightweight Transformer IDS [45]	BoT-IoT, CICIDS2017	99.60	0.990	9.8
GNN-IDS [43]	UNSW-NB15, CICIDS2017	99.50	0.988	14.2
BS-GAT IDS [46]	BoT-IoT, CSE-CIC-IDS2018	99.55	0.989	13.5
Memory-Flow Transformer IDS [47]	CICIDS2017, UNSW-NB15	99.70	0.993	11.2
**Proposed GRU–NTM (Ours)**	**CICIDS2017, UNSW-NB15, BoT-IoT**	**99.98**	**0.996**	**2.3**

### 4.7 Scalability analysis

An important aspect that needs to be taken into account when implementing intrusion detection systems in IoT scenarios in the real world is scalability since the number of interconnected devices and the diversity of traffic on a network can increase at high rates. To assess how the proposed NTM-GRU architecture preserves efficiency and accuracy under these conditions, we conducted a series of experiments in which network size, traffic level, and protocol diversity were progressively varied. To this end, the CICIDS2017 and CSE-CIC-IDS2018 datasets were augmented by appending multiple full-day traffic captures, thus creating datasets which were 1.5, 2, and 3 times bigger than the baseline. Increased heterogeneity was also added with each enlargement, new types of IoT devices like smart sensors, IP cameras, industrial controllers, smart appliance, and communication protocols like HTTP(S), MQTT, CoAP, FTP, Telnet, and proprietary IoT messaging formats. The traffic profiles of these largest datasets varied between low-bandwidth periodic telemetry to high-frequency packet bursts and volumetric floods, providing the possibility of performing a realistic stress test of the model scalability.

The proposed model takes a sequential network traffic data and feeds this data through stacked layers of GRU which will extract short- and mid-term temporal features and then feeds in the information to the NTM component to perform long-term memory activity. The GRU block itself is linear computational complexity with regards to the number of the sequence, however the read/write operations of the memory slots of the NTM are linear with regards to the number of memory slots. This has the effect of the overall complexity being proportional to the product of these two factors. The use of our sparse content-based addressing within the NTM has the property of keeping the implementation efficient, as the number of steps required to complete it remains constant even as the dataset size grows. There are no unnecessary memory operations in the method and the read and write times do not increase with the increase in the number of monitored devices.

Applications of the scalability test were done on the hardware side on both the GPU and CPU platforms. The smallest dataset (baseline) took 2.7 milliseconds of inference time per sample on an NVIDIA RTX 3080 GPU with 10 GB of VRAM, with larger dataset sizes taking slightly longer (3x the baseline size took 8.1 milliseconds per sample), which is well below the 10 milliseconds threshold of real-time inference that is required in Security Operation Centre (SOC) applications. The rise in memory utilisation was only 5.1 GB to 7.8 GB over the same range of scaling, giving a lot of headroom in batch processing. Single-threaded mode the single-threaded baseline inference time is 9.5 milliseconds/sample on Intel 12700K i7 processor and 32 GB RAM, and 27.3 milliseconds/sample on 3x dataset. When multi-threaded execution is enabled, the average per-sample time was cut down to less than 12 milliseconds; this proves the fact that the model can be kept operational despite operating as a CPU-bound system.

Notably, the performance of detection was similar in all the dataset sizes. The accuracy was kept above 99.0 at all scales, the F1-score was at least 0.992 on both known and zero-day classes of attacks and the false positive rate only increased slightly to 0.12 percent at the most enormous scale compared to the baseline rate of 0.08 percent. The above findings validate the fact that the proposed architecture is capable of addressing high-load, heterogeneous network environments without compromising the level of detection capability.

The last argument is that the relatively small extra memory requirements and the almost linear relationship between inference latency and number of added parameters imply that the suggested NTMGRU model can be used in existing SOC pipelines without huge hardware upgrades in the sense of deployment. In edge deployments, the NTM memory size and the quantisation of GRU weights can be reduced to make latency on embedded AI accelerators low. The independence of the architecture in sequence processing enables horizontal scaling across a variety of processing nodes, allowing for the effective management of extreme distributed IoT traffic and eliminating bottlenecks in the context of cloud or ISP-scale monitoring. Such combination of scalability, accuracy, and efficiency makes the given model quite appropriate in the context of large-scale and dynamic intrusion detection environments.

## 5 Resources of time-constrained deployment in scalability

The lack of efficient scaling capabilities when facing a growing amount of network traffic and the capability to operate in real-time in the environments with relatively low computation capacity are among the burning issues when it comes to the deployment of the deep learning-based networks security models. GRU-NTM model proposed has been developed to observe these limitations, and thus, presents a trade-off between performance, efficiency, and flexibility. Its design integrates the time learning of Gated Recurrent Units (GRUs) and the long-term pattern retention and external memory of Neural Turing Machines (NTMs) that together make it more appropriate to scalable networks and applications that require low latencies.

The GRUs are superior in that they are naturally much lighter and even more efficient compared to the conventional LSTM cells thus they present less computational overhead, yet do not compromise with temporal modeling capability. This enables the model to read sequential elements of input data at increased throughput rates with reduced memory overhead, which is a necessity when identifying huge amounts of traffic in distributed infrastructures occurs. Based on our model, the average latency of inference is close to 2.3 milliseconds which proves that it is applicable in real-time analysis in terms of both the packet-level and flow-level. Such a performance measure enables it to be deployed into high-performance enterprise systems and Software-Defined Networks (SDNs), where the ability to make decisions quickly is imperative to deny the spread of malicious activity.

Besides its efficient temporal processing nature, the NTM component comes along with a memory-augmented design that expands its capacity to track low-and-slow or persistent attack traffic throughout a long period. In contrast to classical models which only rely on propagation through the internal state, the NTM allows writing and reading to some external memory matrix, therefore preserving context over long chains of events. It is especially applicable when any attacks can occur over time or repeatedly at spaced intervals, which are typical traits of Advanced Persistent Threats (iv) Advanced Persistent Threats (APTs) and botnet communications nowadays. The GRU-NTM model is an architecture with modularity and lightweight footprint, thus making the model a good candidate for real-time implementation in resource-limited devices i.e. IoT edge nodes, smart gateways, or embedded network appliances. The framework does not need deep and computationally intense convolutional stacks or transformer architectures thus it is less greedy in hardware acceleration and power. In addition, the compatibility of the model with batch-based streaming data pipelines facilitates its operation even in a situation with occasional connectivity, or in a situation with a limited processing window, which may be the most common in fog computing scenarios or mobile security.

## 6 Performance in the noisy and dynamic networks environments

To provide the effectiveness of intrusion detection models in practice, their performance needs to be tested in situations of noisy, unstable, and changeable network conditions. Although some benchmark databases, including UNSW-NB15, BoT-IoT, CICIDS2017, and CSE-CIC-IDS2018, have given an assured scenario to model validation, they cannot effectively model the unpredictable, varying of multiple sources that ring true in live networks. In the real-world, traffic variations like packet sizes, simultaneous legitimate and hostile traffic, encrypted traffic, interprotocol traffic, deformed headers along with background noise smearing benevolent irregularities or physical noise are common features. Such conditions can prove to be a major burden on a model that would be able to draw a line between genuine threats and false alarms.

To overcome this issue, GRU-NTM model was proposed as a dual-mechanism model with GRU layers that efficiently learnt short- and medium-term temporal patterns while a Neural Turing Machine (NTM) was proposed to introduce long-term memory and the capability of anomaly tracking which is essential in high-noise disorders. At inspection, we found that the model had a minimal false positive rate (< 0.4%) on a number of datasets despite having overlaps and unclear actions of attacks. This finding means that there must be high tolerance of noise and the noise-augmented portion certainly aids in making distinctions between benign variations and changing threats given enough time.

Besides, generalization capabilities of the architecture on various datasets are good indicators of a strong architecture. The testing of the model with the real-world datasets of CICIDS2017 and CSE-CIC-IDS2018 possessing the realistic traffic flows and variety of attacks allowed us to be sure that the model shows a consistently high detection and classification accuracy even in the cases of stealthy or slow-evolving patterns of attacks. This effect shows its suitability to use in dynamic operational settings, in which these attackers might employ advanced obfuscation methods, e.g., protocol tunnels, encryption, or traffic objection. In the future we intend on increasing the resilience of the model further by introducing noise-injection methods into the training process. These are random modifications of packet headers, packet loss simulation, and mixing actual benign traffic with adversarial attack sequences to replicate complicated network traffic. The model will also be tested with live-streamed traffic on real networks and software-defined systems giving a real-time possibility to test the model in the conditions of actual workload. These measures will give more information about its flexibility, latency response and accuracy under high entropy, parallel traffic flows and zero-day attacks.

Altogether, the GRU-NTM composite structure is an potentially valuable composition capable of working with realistic, noisy, and changing networks. The low false positive ratio, its adaptive properties enhanced by use of its memory, and reproducibility across datasets, make it a possible and scalable solution that can be implemented in large enterprises, internet-of-things, and cloud-edge cybersecurity systems in which operational uncertainty is the rule, rather than exception.

## 7 Conclusion

The increasing level of sophistication and occurrence of cyber threats, notably DoS and DDoS attacks, pose severe threats to the resiliency and security of contemporary network infrastructures, in general, and the dynamic and resource-limited scenarios of the Internet of Things (IoT), in particular. The ubiquitous threats that are continuously evolving cannot be effectively tackled using traditional IDS software due to its limitations in memory and inability to adapt to changes, resulting from the impact of strict rules that detect objects through specific parameters. We have introduced here a new framework of hybrid deep learning, which goes by the name GRU-NTM, i.e., an integration of the temporal model power of Gated Recurrent Units (GRUs) with the external memory and inference abilities of Neural Turing Machine (NTM). With such a hybrid architecture, this model is capable of modelling both the short- and long-term dependences in network traffic, which also means that it is also expedient at detecting a wide range of intrusions, i.e., those volumetric floods as well as stealthy, slow application-based attacks. The experimentation of GRU-NTM model was conducted on reference datasets (UNSW-NB15, BoT-IoT) and real traffic datasets (CSE-CIC-IDS2018, CICIDS2017) and proved to be the top-performing model with an accuracy of 99.98%, very low false positive rates (below 0.4 percent) and was able to effectively detect zero-day and unseen attacks, recording F1-score above 96 percent.

Besides its accuracy, the model proved viable in real-time deployment, averaging 2.3 ms inference speed and exhibiting wasteful resource usage; alternatives that are likely to meet the demands of next-generation intrusion prevention systems. Introducing interpretability aspects into the system, which would include attention mechanisms and gating structures, would be accompanied by explanations and actionable advice, thereby improving trust and decision support in high-priority Security Operation Centres (SOCs).

Although our solution can alleviate the most significant shortcomings of current IDS models, the methodology can be further refined in the future to optimize performance on edge devices and consider more realistic datasets, as well as incorporate Explainable AI (XAI) models to achieve a higher degree of interpretability. The possibilities of combining the new generation of technologies, such as quantum computers and edge intelligence, will also be explored to enhance scalability and responsiveness. In total, the proposed work can be considered a significant contribution to the field of intelligent intrusion detection, providing the reader with a scalable, adaptable, and high-performance solution to protect ultra-modern and future networked systems against continuously increasing cyber threats.
